# The Burden of Genital Warts in Finland: Cross-Sectional Analysis of the Prevalence and Direct Medical Costs in 2018

**DOI:** 10.3390/vaccines11071202

**Published:** 2023-07-05

**Authors:** Annette Gylling, Kristiina Uusi-Rauva, Iiro Toppila, Eija Hiltunen-Back

**Affiliations:** 1 MSD Finland Oy, 02150 Espoo, Finland; 2 Medaffcon Oy, 02130 Espoo, Finland; kristiina.uusi-rauva@medaffcon.fi (K.U.-R.); iiro.toppila@medaffcon.fi (I.T.); 3 Venereal Diseases Outpatient Clinic, Inflammation Center, Skin and Allergy Hospital, Helsinki University Hospital, 00029 Helsinki, Finland; eija.hiltunen-back@hus.fi

**Keywords:** disease burden, genital warts, human papilloma virus, healthcare resource use, healthcare costs, prevalence, register study

## Abstract

Genital warts (GWs) caused by the human papilloma virus (HPV) are a significant health problem due to high prevalence and rate of recurrence. Bivalent vaccine has been used since the start of the national vaccination program in 2013, making it feasible to study the GW burden in Finland. There is no national and up-to-date information available on the prevalence and the burden of GWs in the various healthcare sectors in Finland. The present study investigated the prevalence, healthcare resource use, and direct medical costs of the treatment of GWs in Finland in 2018 using data in national healthcare registers. GW cases were identified based on diagnoses in public healthcare and GW-related prescription medications. Cost analysis included public healthcare contacts, procedures in private care, and medications. The study showed that approximately 12,000 GWs cases were treated in Finland in 2018. Since less than half of GW diagnoses were recorded in public healthcare registers, determining the exact costs was challenging. The estimated direct treatment costs in 2018 were 2.6 M€, which is higher than the previous estimation in Finland, yet still likely an underestimation of the true burden. These results provide information for the management of the GW burden in Finland.

## 1. Introduction

Genital warts (GWs; *condyloma acuminata*) are wart-like lesions of the anogenital area caused by human papillomavirus (HPV) [1,2]. GWs are one of the most common sexually transmitted infections [3,4]. In fact, the World Health Organization has lined elimination of GWs, among other two sexually transmitted infections, as one of its strategies [5]. In European countries, the annual incidence of new cases is estimated to be 118–170 cases per 100,000 people [4]. The incidence is highest among 20–29-year-olds [4,6,7]. In this age group, the negative effects of GWs on sexuality and relationships can significantly reduce the quality of life [8]. The global annual incidence of new and recurrent GWs is estimated to be 160–289 cases per 100,000 persons. Approximately 118–205 cases per 100,000 persons are new GW diagnoses [4]. Ninety percent of GWs are caused by HPV low-risk virus types 6 and 11 infections, which can be reduced with quadri- and 9-valent HPV vaccines. Significant reductions and near elimination of GWs have been seen early on in countries using vaccines containing GW protection in the national vaccination program. The quadri- and 9-valent vaccine is used in national immunization programs in most European countries and elsewhere [9,10,11,12,13,14,15,16].

Although GWs are generally considered a non-serious disease, it is nevertheless a significant health problem. The prevalence, recurrence risk, re-infections, and the treatment of prolonged cases, as well as the effects on the quality of life cause a significant burden from the perspective of both patients and healthcare. The incubation period of GWs from infection to possible symptoms is estimated to be approximately 1–8 months [2]. Condom use does not completely protect against GWs, because the virus can also be on the skin not covered by the condom. Eighty to ninety percent of anogenital HPV infections clear within a year, however, in 10% the virus remains active. Most anogenital HPV infections are asymptomatic, and thus a person can transmit the virus without knowing it. The diagnosis of GW is based on a clinical examination by a physician [1,2]. Visible lesions in GW are treated either with local medical treatments carried out by the patient at home or removed by freezing (cryo) and laser treatments performed by a healthcare professional [1,2]. Irrespective of treatment type, the tendency of GWs to recur is high, as the treatments do not destroy the virus [1,2,17,18].

In Finland, most GW cases are diagnosed in public primary healthcare sector, which has a significant role especially in municipalities where secondary healthcare services are not available [1,6,7]. The choice of treatment depends on the location and the number of warts, the equipment available, and patient’s ability to adhere to the treatment. The two main pharmacological treatments for GWs in Finland are imiquimod and podophyllotoxin [1]. The availability and reimbursement status of the GW-related medications have varied over the years in Finland, which may have also had an impact on treatment practices. Different forms of treatment are often used during a treatment episode. The advantage of self-applied treatment is the possibility to choose time that suits the patient. Repeated treatments carried out at healthcare clinics during office hours involve absences from work or studies.

Unlike in many other European countries, the national HPV vaccination program in Finland does not currently use a vaccine that contains GW-causing HPV types [19]. In addition, GWs are not among infectious diseases monitored under the infectious disease legislation, so there is no comprehensive national and up-to-date information on the prevalence and, thus, the actual disease burden of GWs in Finland. According to a previous study, an average of 6 800 new cases of GWs were diagnosed in Finland per year between 2000 and 2014, and the annual direct GW-related treatment costs, reported at the price level of 2016, were estimated to be 2.034 million euros [7]. According to another Finnish study, the treatment of GWs in women, reported at the price level of 2010, caused an average annual cost of about 0.9 million euros [20].

The aim of this work was to assess the prevalence and the up-to-date burden of GWs in Finland. The number of patients and burden associated with the direct medical costs in the treatment of GWs, including non-reimbursed medications, were assessed for the year 2018. The treatment costs of GW were considerably higher compared with the previous estimations from Finland.

## 2. Materials and Methods

### 2.1. Study Design, Permits, and Data Sharing

The present non-interventional, retrospective cross-sectional study was conducted in accordance with the Declaration of Helsinki. Data was extracted from healthcare registers and linked using personal identity numbers of the subjects and aggregated by the Finnish Social and Health Data Permit Authority, Findata, in accordance the Act on Secondary Use of Health and Social Data 552/2019, Finland. Data request was approved by Findata (THL/4994/14.01.00/2020). Results with low subject numbers were masked due to the anonymity requirement of Findata (n < 5). The second lowest n number (and related data) was also masked, or one n number was provided as a range, as feasible, to ensure that the masked numbers cannot be deduced based on other available numbers in the data set. Therefore, some n numbers in the publication were rounded to the nearest tenth.

### 2.2. Eligibility Criteria

Prevalent GW cases were identified in the national registers of outpatient prescription medications and public healthcare contacts, visits, and treatment periods. Each subject was considered only once. Inclusion criteria were as follows: at least one ICD-10 or ICPC-2 diagnosis for GW as a main diagnosis (Table 1, inclusion criteria) in the Register of Primary Health Care Visits (Finnish Institute for health and welfare, THL) or in respective secondary/tertiary care register, i.e., the Care Register for Health Care (THL), or, at least one record of GW-related medication purchases in the Prescription Center (Social Insurance Institution, SII) or in the reimbursements of the SII (Table 1, inclusion criteria), in 2018. The two data sources of outpatient prescription medications overlap in terms of reimbursed medications. The Prescription Center has data on all outpatient prescriptions and corresponding purchases since 2017. 

Subjects who, in addition to an imiquimod purchase, had one of the exclusion diagnoses in Table 1 recorded as a main diagnosis in the Register of Primary Health Care Visits (data from 2011 onward) or the Care Register for Health Care (data from 2010 onward), either on the day of the imiquimod purchase or before, were excluded from the analyses. 

The clinical expert of the study validated all the codes utilized in the cohort formation and data extraction.

### 2.3. Data Sources, Patient Characteristics, and Healthcare Resource Use

Data on purchased GW-related prescription medications (Table 1), public healthcare contacts, visits, and treatment periods with GW as a main diagnosis (Table 1), and reimbursed procedures in private care (Table 2) in 2018, were originally extracted from the following registers: the register of medications reimbursed from health insurance (SII), the Prescription Center (SII), the Register of Primary Health Care Visits (THL), and the Care Register for Health Care (THL). The Prescription Center was primarily utilized in the analysis of the GW medications.

Age of the subjects was defined as the date of the first event that met the inclusion criteria (index).

The costs of reimbursed procedures in private care and outpatient prescription medications in 2018 were directly obtained from the registers. The costs of public healthcare resource use (HCRU) in 2018 were determined based on the aggregate data on the number of events in the study cohort in 2018 and publicly available unit costs of the healthcare in 2017 [21], which were scaled to 2018 price level using a price index of public expenditure [22]. The unit costs of public healthcare include average costs of examinations, procedures, inpatient medications, and overheads estimated for each unit and the type of contact/visit, and occupational group/specialty field. In the analysis, primary care contacts, which were not possible to price with one specific unit cost in [21], were included with a minimum estimate, i.e., the lowest unit cost available for each type of profession/contact in [21] was used. A unit cost corresponding to a primary care doctor’s visit (83 euros per case in 2017 unit cost data) was added for cases, that did not have public healthcare contacts recorded for a GW diagnosis in 2018, but that were identified based on record(s) of GW medication(s).

## 3. Results

### 3.1. Prevalence of Genital Warts in 2018

**Figure 1:**
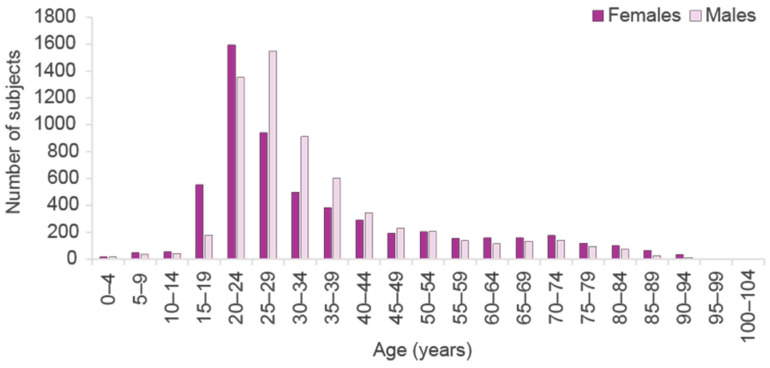
The number of genital wart cases per age group in Finland in 2018.

There were 11,919 unique GW cases in Finland in 2018. The number includes both new and previously treated, prevalent subjects. Of these, 48% were females (n = 5728/11,919) and 52% males (n = 6191/11,919). Mean age at index was 35 years (standard deviation, SD: 17 years). GWs were as expected most common in 20–29-year-olds with females peaking at younger age (Figure 1). Overall, approximately 25% (n = 2946/11,919) of the cases represented 20–24-years-olds, while 21% (n = 2489/11,919) were 25–29 years of age at index. It should be noted that older age groups may also include some individuals with purchases of imiquimod also for other indications.

### 3.2. Health Care Resource Use and Direct Medical Care Costs of Genital Warts in 2018

**Figure 2:**
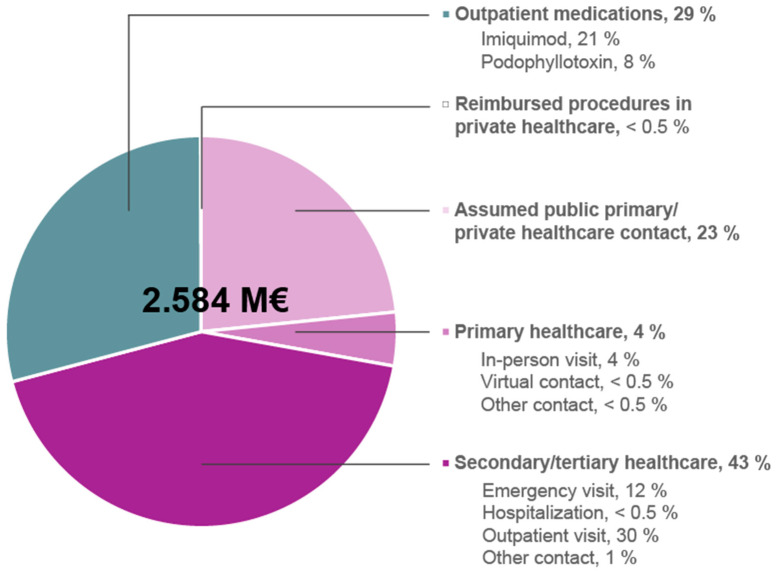
Total direct medical care costs of the treatment of genital warts in Finland in 2018. Public healthcare costs include average costs of examinations, procedures, inpatient medications, and overheads. The costs of the procedures in private care include only those procedures that were reimbursed for at least eight people in the study cohort. Assumed contact included pharmacologically treated subjects who had no records on public healthcare contacts.

HCRU analysis included reimbursed procedures in private care, outpatient prescription medications, and public HCRU in 2018. The estimated direct medical care costs of GWs in 2018 in Finland were 2.584 million euros (Figure 2). A majority, ≈71% of the total costs (1.833 million euros), were due to outpatient and inpatient HCRU, while ≈29% of the costs (751,000 euros) were explained by the utilization of the outpatient prescription medications (Figure 2). The number of reimbursed procedures performed in private healthcare was negligible in the assessed GW cohort (Figure 2). The number of procedures performed in public healthcare was also minimal (data not shown; the costs are included in the unit costs of public HCRU).

Table 3 shows the breakdown of the costs and respective subject and event numbers. The majority, 82% (n = 9721/11,919) of the GW cases in 2018 were treated pharmacologically. Of these, 37% (n = 3603/9721) had purchased prescription medication for GW without obtaining a reimbursement (i.e., records in Prescription Centre, but none in the SII’s reimbursements). Overall, podophyllotoxin was slightly more commonly utilized (n = 7063 purchases, n = 5612 subjects) compared to imiquimod (n = 6545 purchases, n = 4703 subjects), with 6% (n = 594/9721) of the subjects having purchased both medications in 2018 (Table 3). A majority, 73%, of the medication costs were, however, due to imiquimod, while 27% were related to podophyllotoxin (Table 3).

Based on public healthcare contacts recorded for GW as a main diagnosis, the main driver in the public HCRU for GW in 2018 were outpatient visits in secondary/tertiary care (3112 visits per 11,919 GW subjects), followed by in-person visits in primary care (2225 visits per 11,919 GW subjects) and “emergency visits” in secondary/tertiary care (998 visits per 11,919 GW subjects; including appointments not booked beforehand; Table 3). Altogether, 4215 and 2604 inpatient/outpatient contacts or hospitalization days in total were recorded for secondary/tertiary and primary public healthcare in 2018, respectively, among identified 11,919 GW cases (Table 3). However, the number of recorded public primary care contacts is likely an underestimation as up to 60% (n ≈ 7190/11,919) of the cases had no records for GW as a main diagnosis in public healthcare in 2018, but only imiquimod and/or podophyllotoxin purchases. These cases likely represent pharmacologically treated patients who received a prescription for GW in private healthcare, whose GW-related healthcare visit took place at the end of 2017, or whose GW diagnoses at public healthcare were not comprehensively recorded. Therefore, one public primary care visit was added per such subject in the cost analysis (Table 3 and Figure 2). It should be noted that some of these subjects may have been diagnosed in private care.

## 4. Discussion

The present study is aiming to contribute to update the knowledge and information on the economic and clinical burden of the treatment of GWs in Finland. In the absence of surveillance data, the burden of GWs and its distribution in different healthcare sectors in Finland can be estimated from the register data on outpatient medications and healthcare utilization. Data can be extracted from multiple national registers and linked using personal identity numbers. According to previous Finnish register data-based estimate, approximately 6800 new/incident cases of GWs were diagnosed in Finland each year [6,7]. Our study provides novel information on the total number of subjects treated for GW per year by demonstrating that there were a total of nearly 12,000 persons per ca 5.5 million inhabitants, who bought GW medications or used public healthcare services with GW as a main diagnosis in 2018. The number includes both new/incident and recurrent/prevalent subjects in 2018, and, thus, is not directly comparable with the previous data on 6800 incident subjects per year. These studies highlight the role and quality of Finnish social and healthcare register data in regular evaluation of the burden of infectious diseases in Finland.

Distribution of cases by sex and age group in 2018 are roughly in line with previous observations [6,7]. The age difference at the GW diagnosis between men and women is at least partly due to later sexual debut in men. The number of GW patients observed in primary healthcare in the present study was surprisingly small, which may partly be due to possibly missing diagnosis records. More comprehensive records of GW diagnoses in healthcare would enhance the assessment of the actual burden. 

The estimated economic burden of the treatment of GWs in 2018 in Finland was 2.6 million euros, including only direct medical costs. The true burden is likely higher due to possibly missing records on GW diagnoses in public healthcare and because the study did not include private healthcare visits. Therefore, the GW cohort of the present study did not include those potential GW cases diagnosed in private healthcare who only had contacts with private care and who did not have purchases of prescription medications for GW in 2018. Moreover, only some of the podophyllotoxin products available in Finland in 2018 were reimbursed by the SII. After 2018, none of the podophyllotoxins have been reimbursable. Podophyllotoxins are currently only available on temporary special permit and are therefore more expensive for patients. The increased prices and interruptions in availability of medications have probably impacted on treatment choices. 

The estimated direct, annual economic burden of the treatment of GWs in Finland was 27% higher than previously reported (2.0 million euros) [7]. The difference likely results from several potential factors, including changes in healthcare costs in general, patient numbers, and treatment practice across years, as well as from differences in the study set up. The unit costs of healthcare have changed in one way or the other depending on the healthcare type in question. The costs (before index scaling) estimated based on 2017 unit costs were only ca 13% higher than the costs analyzed using 2011 data [23]. This suggests that the observed increase in the economic burden of GW is solely not explained by the overall higher unit costs of healthcare or index scaling. In contrast to previous study, the present estimation also included non-reimbursed prescription medications as such data has since become available. It is noteworthy that approximately a third of the pharmacologically treated cases had purchased prescription medication for GW without obtaining a reimbursement, and if only reimbursed purchases would have been included, the costs of the medications in the treatment of GW in 2018 would have been approximately 27% lower than the costs of all purchases (data not shown). For comparison among Nordic countries, in Sweden in 2009, i.e., before the national HPV immunization program, total costs in up to 45 years-olds GW cases were 9.8 million euros, of which 8.2 million euros represented direct medical costs [24]. In Norway and Denmark, where the populations are roughly the same size as in Finland, the total costs were estimated to be approx. 240–300 euros per case [25,26]. Considering the incidence rates in the two countries in 2009 before the HPV vaccination program [27], the total annual costs in 12–35-year-olds would have been at least 3 million euros. However, the studies cannot be directly compared with each other due to different study settings.

Limitations of the present study include potentially older subjects who have used imiquimod for other indications than GW, which may lead to potential, but likely slight, overestimation of the number of GW cases and/or GW medication costs. These subjects may have not been comprehensively excluded in the cohort formation due to missing records on other imiquimod indications. The present study is a single-year study, which is prone to potential year-to-year variation.

As a conclusion, we set out to study the burden of GW in Finland because it is one of the few countries left in Europe where this type of study is still feasible both due to the vaccine in the national vaccination and the healthcare registers. The burden associated with the treatment of GWs is considerable in Finland, where a vaccine protecting against GWs is currently not included in the national vaccination program [19]. Use of HPV vaccines that include protection against GWs in national immunization programs have been shown to reduce the burden of GWs in many countries [14,28,29,30,31,32,33]. Understanding the full GW burden in Finland, and to discuss the benefits of reducing GWs is important even after a decade of a well-working vaccination program. Reducing GW saves healthcare resources sooner than the reduction of HPV-related cancers and precancers, but more importantly spares primarily young people from both unnecessary treatment rounds and the psychological burden associated with the disease.

## Figures and Tables

**Table 1:** Diagnosis codes and medications utilized in eligibility criteria.

ICPC-2	ICD-10	ATC Code (Effective Substance)
**Inclusion criteria:**		
Y76 (Condylomata acuminata male)	A63.0 [Anogenital (venereal) warts]	ATC D06BB10 (imiquimod)
X91 (Condylomata acuminata female)		ATC D06BB04 (podophyllotoxin)
**Exclusion criteria:**		
S77 (Malignant neoplasm of skin)	C44 (Other and unspecified malignant neoplasm of skin)	
X77 (Malignant neoplasm genital female other)	C51 (Malignant neoplasm of vulva)	
S80 (Solar keratosis/sunburn)	L57.0 (Actinic keratosis)	
S79 (Neoplasm skin benign)	D04 (Carcinoma in situ of skin)	

Abbreviations: ATC, Anatomical Therapeutic Chemical Classification.

**Table 2:** Procedure codes for genital wart treatments.

NCSP Code	Description
KGD10	Destruction of lesion of penis
LFB10	Excision of lesion of vulva or perineum
LFB20	Destruction of lesion of vulva or perineum
KDD32	Urethroscopic destruction of tumor of urethra
LDB00	Excision of lesion of cervix uteri
LDB20	Electrocoagulation or laser therapy of cervix uteri

Abbreviations: NCSP, the Nordic Classification of Surgical Procedures.

**Table 3:** Total direct medical care costs of the treatment of genital warts in Finland in 2018, stratified by resource type.

	Number of Subjects ^1^	Subcategory	Number of Subjects Per Event ^1^	Number of Events ^1,2^	Total Costs (€) ^1^
**Secondary/** **tertiary** **public healthcare**	≈2220	Emergency care visit ^3^	NA ^4^	997.5	315,179
		Hospitalization days		5.0	3851
		Outpatient visit		3111.5	763,927
		Other speciality care contact		100.5	30,060
		**Total costs**	**1,113,017**		
**Primary** **public healthcare**	2773	In-person visit	NA ^4^	2225.0	102,784
		Virtual contact		190.0	5742
		Other		188.5	5892
		**Total costs**	**114,418**		
**Assumed** **public primary/** **private healthcare contact ^5^**	≈7190	-	7190	7190	604,532
**Reimbursed** **procedures at** **private sector**	≈10–16	KGD10	X	X	X
		LFB10	8	8	1155
		LFB20	X	X	X
		KDD32	0	0	0
		LDB00	0	0	0
		LDB20	0	0	0
		LEB30	0	0	0
		**Total costs ^6^**	**1155**		
**Outpatient** **prescription** **medications**	9721	Imiquimod	4703	6545	547,953
		Podo- phyllotoxin	5612	7063	203,391
		**Total costs**	**751,344**		
**Total costs**	**2,584,465**				

^1^ Results are based on aggregate data where low subject numbers (n < 5) were masked (“X”) due to the anonymity requirement of the data authority. The second lowest n number (and related data) was also masked, or one n number provided as a range, as feasible, to ensure that the masked numbers cannot be deduced based on other available numbers in the data set. Therefore, some n numbers in the publication were rounded to the nearest tenth. ^2^ In the public healthcare resource use analysis, masked event numbers were imputed with a value of 2.5, thus resulting in half digits for contacts. ^3^ Including appointments not booked beforehand and potential visits, if any, occurring after standard office hours. ^4^ NA, not available. Subject numbers were extracted in more detailed level (see the methods). Thus, pooling of unique subjects not possible for categories presented in the table. ^5^ 60% (n ≈ 7190/11,919) of the cases had no records for GW as a main diagnosis in public healthcare in 2018, but only imiquimod and/or podophyllotoxin purchases. Therefore, one unit cost of 83€ per subject was applied. See the methods. ^6^ Due to the anonymity requirement and masking, the costs of the procedures in private care include only those procedures that were reimbursed for at least eight people in the study cohort.

## Data Availability

Extracted individual-level data cannot be shared. Only the personnel of each data source and the Finnish Social and Health Data Permit Authority, Findata, had access to the individual-level data and can grant rights to third parties to use the data in accordance with the Act on Secondary Use of Health and Social Data.
